# Carbon disulfide – Determination of carbon disulfide in workplace air using headspace gas chromatography (headspace-GC-FPD): Air Monitoring Method – Translation of the German version from 2024

**DOI:** 10.34865/am7515e10_2or

**Published:** 2025-06-30

**Authors:** Andreas Grill, Claus-Peter Maschmeier, Ralph Hebisch, Uta Lewin-Kretzschmar, Andrea Hartwig, MAK Commission

**Affiliations:** 1 Kelheim Fibres GmbH Regensburger Str. 109 93309 Kelheim Germany; 2 Federal state Saxony-Anhalt Gebrüder-Bethmann-Str. 18 06862 Dessau-Roßlau Germany; 3 Federal Institute for Occupational Safety and Health (BAuA) Friedrich-Henkel-Weg 1–25 44149 Dortmund Germany; 4 German Social Accident Insurance, Institution for the raw materials and chemical industry, Prevention - Department of Hazardous Substances, Biological Agents and Analytical Chemistry Kurfürsten-Anlage 62 69115 Heidelberg Germany; 5 Institute of Applied Biosciences. Department of Food Chemistry and Toxicology. Karlsruhe Institute of Technology (KIT) Adenauerring 20a, Building 50.41 76131 Karlsruhe Germany; 6 Permanent Senate Commission for the Investigation of Health Hazards of Chemical Compounds in the Work Area. Deutsche Forschungsgemeinschaft, Kennedyallee 40, 53175 Bonn, Germany. Further information: Permanent Senate Commission for the Investigation of Health Hazards of Chemical Compounds in the Work Area | DFG

**Keywords:** Schwefelkohlenstoff, Luftanalysen, Analysenmethode, Arbeitsplatzmessung, Gefahrstoff, Headspace-Gaschromatographie, flammenphotometrische Detektion, Headspace-GC-FPD, Aktivkohle, Flüssigdesorption, carbon disulfide, air analyses, analytical method, workplace measurement, hazardous substance, headspace gas chromatography, flame photometric detection, headspace-GC-FPD, activated charcoal, liquid desorption

## Abstract

The working group “Air Analyses” of the German Senate Commission for the Investigation of Health Hazards of Chemical Compounds in the Work Area (MAK Commission) developed and verified the presented analytical method. It is used to determine the levels of carbon disulfide [75-15-0] that occur in the workplace air. The method covers concentrations in the range from one tenth to twice the current German occupational exposure limit value (OELV) of 30 mg/m^3^. It is also suitable for monitoring compliance with the MAK value of 16 mg/m^3^ and the short-term exposure limit (STEL; excursion factor 2). Samples are collected by drawing a defined volume of air through a sampling tube filled with activated charcoal using a flow regulated pump at a maximal volumetric flow rate of 0.333 l/min. Exposure during the shift is measured with a sampling period of 2 hours (up to 8 hours, depending on the volumetric flow) and the short-term exposure with a period of 15 minutes. The carbon disulfide adsorbed to the activated charcoal is extracted with toluene and analysed by headspace gas chromatography with flame photometric detection. The quantitative determination is based on multiple-point calibrations with an internal standard. A relative limit of quantification (LOQ) of 0.5 mg/m^3^ is obtained for an air sample volume of 40 litres and an application volume of 18 ml. As the relative LOQ for a sample volume of 5 litres is below 5 mg/m^3^, the STEL can also be measured. The recovery, which has to be considered for the calculation of the results, is approx. 70% and the expanded uncertainty is below 22% for a sampling period of 2 hours and below 23% for a period of 15 minutes.

**Table:** 

**Method number**	3
**Application**	Air analysis
**Analytical principle**	Headspace gas chromatography with flame photometric detection (headspace-GC-FPD)

## 1 Characteristics of the method

**Table:** 

**Precision:**	Coefficient of variation:	*s* = 2.4 to 6.7%
	Expanded uncertainty:	*U* = 21.2 to 21.8%
	in the range of 3.0 to 45 mg/m^3^ and n = 5	
**Limit of quantification:**	Absolute:	0.5 mg/l
	Relative:	0.5 mg/m^3^ for an air sample volume of 40 l and an application volume of 18 ml 4 mg/m^3^ for an air sample volume of 5 l for short-term measurements and an application volume of 18 ml
**Recovery:**	*η* = about 70% (included in the results calculation; must be determined)	
**Sampling recommendations:**	Sampling period:	2 h to 8 h
	Air sample volume:	40 l
	Volumetric flow rate:	0,333 l/min to 0,083 l/min
	For short-term measurements:	15 min; 0,333 l/min

## 2 Description of the substance

### Carbon disulfide [75-15-0]

Carbon disulfide (formula: S=C=S, CS_2_, also known as carbon bisulfide) is a colourless, highly refractive liquid. Pure carbon disulfide has a pleasant, ether-like odour. In general, this odour can no longer be perceived if impurities are present. The unpleasant odour that is sometimes noticeable is caused by small amounts of other sulfur compounds. Carbon disulfide has a very low flash point and forms explosive vapour-air mixtures in concentrations of 0.6 to 60% (v/﻿v) (RÖMPP-Redaktion and Sitzmann 2009).

Large quantities of carbon disulfide are used in the production of cellulose fibres from pulp. The pulp is first treated with sodium hydroxide to form alkali cellulose. The alkali cellulose then reacts with carbon disulfide to form xanthate, which is soluble in sodium hydroxide, after oxidative degradation. This produces a cellulose solution, also known as viscose, which is spun to regenerated cellulose in coagulation baths containing sulfuric acid. Carbon disulfide is also used to control the grape phylloxera in viniculture, as a reagent in synthesis reactions and as a solvent in infrared spectroscopy. It is furthermore a good solvent for fats, resins, rubber and vulcanised rubber (IFA 2024; RÖMPP-Redaktion and Sitzmann 2009).

Carbon disulfide has an occupational exposure limit value (OELV) of 30 mg/m^3^ (10 ml/m^3^). Its short-term concentration has been assigned to Peak Limitation Category II with an excursion factor of 2 (AGS 2024). Carbon disulfide is included in the List of MAK and BAT Values with a MAK value of 16 mg/m^3^, which is about half the OELV (DFG 2024), and has been assigned to Peak Limitation Category II with an excursion factor of 2. The data for carbon disulfide are listed in Table 1.

**Tab. 1:** Substance data for carbon disulfide (IFA 2024)

Name	Carbon disulfide
CAS No.	75-15-0
Molar mass [g/mol]	76.14
Physical state at 20 °C	liquid
Density at 20 °C [g/cm^3^]	1.26
Vapour pressure at 20 °C [hPa]	395
Melting point [°C]	–112
Boiling point at 1013 hPa [°C]	46
Flash point [°C]	< –20
Assessment criteria	
OELV, Germany (AGS 2024)	30 mg/m^3^
MAK value, Germany (DFG 2024)	16 mg/m^3^

## 3 General principles

This analytical method is used to determine carbon disulfide in the workplace air in a concentration range from 0.1 times to twice the currently valid OELV of 30 mg/m^3^ (AGS 2024). The method is also suitable for monitoring compliance with the peak limitation with the excursion factor of 2 (AGS 2024; DIN 2021 a). The limit of quantification of the method is low enough to use the MAK value of 16 mg/m^3^ as an assessment criterion.

Samples are taken by drawing a defined volume of air from the breathing zone through an activated charcoal tube (type B/G) using a pump. Liquid desorption is performed with toluene, followed by headspace gas chromatography and flame photometric detection (headspace GC-FPD). The quantitative evaluation is based on a multiple point calibration with internal standard (ISTD, thiophene).

## 4 Equipment, chemicals and solutions

### 4.1 Equipment

For sampling:

- Pump for personal and stationary sampling, suitable for a flow rate between 0.083 l/min and 0.333 l/min (e.g. SG350ex, from DEHA Haan & Wittmer GmbH, 71296 Heimsheim, Germany)
- Activated charcoal tubes, type B/G (e.g. from Drägerwerk AG & Co. KGaA, 23558 Lübeck, Germany)
- Tube holder with suitable dimensions (e.g. Art. No. DH520060, from DEHA Haan & Wittmer GmbH, 71296 Heimsheim, Germany)
- Silicon tubing and tube connector to connect the pump and the tube holder
- Volumetric flow meter (e.g. Gilian Gilibrator 3, from Sensidyne LP, St. Petersburg, FL, USA, sold by DEHA Haan & Wittmer GmbH, 71296 Heimsheim, Germany)

For sample preparation and the analytical determination:

- Headspace vials (e.g. crimp neck vial ND20, from Th. Geyer GmbH & Co. KG, 71272 Renningen, Germany)
- Cap (aluminium/silicone) (e.g. crimp cap ND20, from Th. Geyer GmbH & Co. KG, 71272 Renningen, Germany)
- Glass cutter (e.g. from Carl Friedrich Usbeck KG, 42477 Radevormwald, Germany)
- Volumetric flasks made of glass (25 to 200 ml) with glass stoppers (e.g. Blaubrand, from Brand GmbH + CO KG, 97877 Wertheim, Germany)
- Bulb pipettes made of glass (1 to 10 ml) (e.g. Blaubrand, from Brand GmbH + CO KG, 97877 Wertheim, Germany)
- Disposable syringes, 20 ml, made of polyethylene with suitable cannulas
- Analytical balance (e.g. A200S, from Sartorius AG, 37079 Göttingen, Germany)
- Gas chromatograph with a CAP inlet, deactivated liner and flame photometric detector in addition to control and analysis software (e.g. Clarus 590, from PerkinElmer LAS Germany GmbH, 63110 Rodgau, Germany)
- Headspace sampler (e.g. Turbomatrix 40, from PerkinElmer LAS Germany GmbH, 63110 Rodgau, Germany)
- Separation column GS-Q 30 m × 0.53 mm (e.g. J&W, from Agilent Technologies Deutschland GmbH, 76337 Wald﻿bronn, Germany)

### 4.2 Chemicals

- Toluene, ≥ 99.9% (e.g. from Merck KGaA, 64271 Darmstadt, Germany)
- Carbon disulfide, ≥ 99.90% (e.g. from Honeywell International Inc., Morristown, NJ, USA). Two separate batches are required for the calibration and control standards.
- Thiophene, for synthesis, 99.9% (e.g. from Merck KGaA, 64293 Darmstadt, Germany)
- Helium 5.0 (carrier gas) (e.g. from Air Liquide Deutschland GmbH, 40476 Düsseldorf, Germany)
- Hydrogen 5.0 (e.g. from Air Liquide Deutschland GmbH, 40476 Düsseldorf, Germany)
- Synthetic air 5.0 (hydrocarbon-free) (e.g. from Air Liquide Deutschland GmbH, 40476 Düsseldorf, Germany)
- Test gas, 30 mg CS_2_/m^3^ in air (e.g. from Air Liquide Deutschland GmbH, 40476 Düsseldorf, Germany)

### 4.3 Solutions

The following solutions were prepared using the chemicals listed in Section 4.2. All solutions must be stored in the refrigerator:

**Thiophene stock solution:** (5 g thiophene/l in toluene)

0.50 g of thiophene are weighed and placed into a 100-ml volumetric flask. The flask is filled to the mark with toluene and then shaken. The solution can be used for 1 year if stored in the refrigerator.

**Thiophene standard solution:** (250 mg thiophene/l in toluene)

10 ml of the thiophene stock solution are added to a 200-ml volumetric flask containing about 100 ml of toluene. The volumetric flask is filled to the mark with toluene and then shaken. The solution can be used for 6 months if stored in the refrigerator.

**Carbon disulfide stock solution:** (5 g CS_2_/l in toluene)

0.50 g of carbon disulfide are weighed and placed into a 100-ml volumetric flask. The flask is filled to the mark with toluene and then shaken. The solution can be used for 1 year if stored in the refrigerator.

A second, analogue stock solution is prepared for the control standard using a separate batch of carbon disulfide.

**Calibration solution:** (250 mg CS_2_/l in toluene)

10 ml of the carbon disulfide stock solution are added to a 200-ml volumetric flask containing about 100 ml of toluene. The volumetric flask is filled to the mark with toluene and then shaken. The solution can be used for 1 month if stored in the refrigerator.

**Control solution:** (250 mg CS_2_/l in toluene)

5 ml of the carbon disulfide stock solution that was prepared for the control solution are added to a 100-ml volumetric flask containing about 50 ml of toluene. The volumetric flask is filled to the mark with toluene and then shaken. The solution can be used for 1 month if stored in the refrigerator.

**Blank solution:** (25 mg thiophene/l in toluene)

5 ml of the thiophene standard solution are added to a 50-ml volumetric flask containing about 25 ml of toluene. The volumetric flask is filled to the mark with toluene and then shaken. The blank solution is prepared fresh every working day.

### 4.4 Calibration and control standards

The calibration standards are prepared by diluting the calibration solution with toluene as specified in Table 2. For this purpose, the volumes given in Table 2 are added by bulb pipette to ten 100-ml volumetric flasks, each containing about 50 ml of toluene. The volumetric flasks are then filled to the mark with toluene and shaken thoroughly.

The activated charcoal from the adsorption layer of a type B/G tube is placed into a crimp neck vial and covered with 20 ml of the respective calibration standard. The vials are then sealed and let stand for 1 hour at room temperature.

5 ml of these solutions are transferred to 25-ml volumetric flasks by disposable syringe. 2.5 ml of the thiophene standard solution are added to each flask. The flasks are filled to the mark with toluene and then shaken thoroughly. 5 ml of these calibration samples are placed into headspace vials and analysed under the conditions listed in Section 6.

**Tab. 2:** Preparation and concentrations of the calibration standards and samples

**Calibration standard**	**1**	**2**	**3**	**4**	**5**	**6**	**7**	**8**	**9**	**10**
V (calibration solution) [ml]	1	2	3	4	5	6	7	8	9	10
*c* (calibration standard) [mg/l]	2.50	5.00	7.50	10.0	12.5	15.0	17.5	20.0	22.5	25.0
*c* (calibration sample) [mg/l]	0.500	1.00	1.50	2.00	2.50	3.00	3.50	4.00	4.50	5.00
*ρ* (CS_2_) [mg/m^3^]^a)^	6.25	12.5	18.8	25.0	31.3	37.5	43.8	50.0	56.3	62.5
*ρ* (CS_2_) [mg/m^3^]^b)^	1.25	2.50	3.75	5.00	6.25	7.50	8.75	10.0	11.3	12.5
*ρ* (CS_2_) [mg/m^3^]^c)^	0.625	1.25	1.88	2.50	3.13	3.75	4.38	5.00	5.63	6.25

^a)^ for a sampling volume of 40 l (120 min at 0.333 l/min) and an application volume of 1 ml

^b)^ for a sampling volume of 40 l (120 min at 0.333 l/min) and an application volume of 5 ml

^c)^ for a sampling volume of 40 l (120 min at 0.333 l/min) and an application volume of 10 ml

The calibrated range is from 1.3 to 13 mg/m^3^ for a sampling volume of 40 l (120 min, 0.333 l/min) and 5 ml of desorption solution. The calibration range decreases proportionately for an application volume of 10 ml and is between 0.63 and 6.3 mg/m^3^. The calibration range increases for an application volume of 1 ml and is between 6.3 and 63 mg/m^3^ (see Table 2).

By varying the application volume, the calibration range that is appropriate for the expected concentration can be chosen. With suitable dilutions, it is possible to analyse volumes that are close to or even exceed the OELV. This covers the required range of one tenth to twice the currently valid OELV.

Control standards are prepared fresh every working day using dilutions of the control solution in toluene as specified in Table 3. For this purpose, the volumes given in Table 3 are added by bulb pipette to two 50-ml volumetric flasks containing about 25 ml of toluene. The volumetric flasks are filled to the mark with toluene and then shaken thoroughly.

The activated charcoal of the adsorption layer (the larger layer of activated charcoal) of a type B/G tube is transferred to a crimp neck vial and covered with 20 ml of the respective control standard. The vials are sealed and let stand for 1 hour at room temperature.

5 ml of these solutions are placed into 25-ml volumetric flasks by disposable syringe. 2.5 ml of thiophene standard solution are added to each flask. The volumetric flasks are filled to the mark with toluene and then shaken thoroughly. 5 ml of each control sample are placed into headspace vials and analysed under the conditions given in Section 6.

**Tab. 3:** Preparation and concentrations of the control standards and samples

**Control standard**	**1**	**2**
V (control solution) [ml]	1	4
*c* (control standard) [mg/l]	5.00	20.0
*c *(control sample) [mg/l]	1.00	4.00
*ρ* (CS_2_) [mg/m^3^]^a)^	12.5	50.0
*ρ* (CS_2_) [mg/m^3^]^b)^	2.50	10.0
*ρ* (CS_2_) [mg/m^3^]^c)^	1.25	5.00

a) for a sampling volume of 40 l (120 min at 0.333 l/min) and an application volume of 1 ml

b) for a sampling volume of 40 l (120 min at 0.333 l/min) and an application volume of 5 ml

c) for a sampling volume of 40 l (120 min at 0.333 l/min) and an application volume of 10 ml

## 5 Sampling and sample preparation

### 5.1 Sampling

**Fig. 1:**
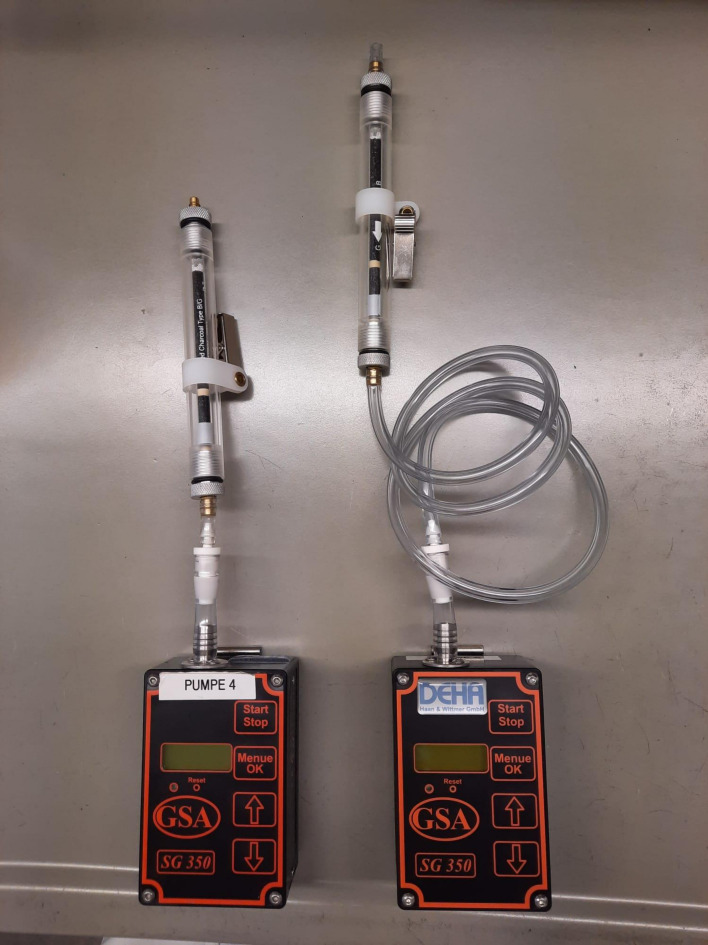
Assembled sample carriers, at left for stationary measurements, at right for personal measurements. The assembly is made up of a sampling tube (type B/G) in a tube holder with a connecting tube and a sampling pump (SG350ex)

A glass cutter is used to cut open the activated charcoal tube immediately before the start of sampling. The charcoal tube is mounted tightly in the tube holder and the tube holder is connected to the pump with a tubing of suitable length (personal or stationary; see Figure 1, stationary at left, personal at right). The direction of air flow should be checked. The larger layer of activated charcoal is used as the adsorption layer (type G).

A representative sample carrier from the same production batch is used to set the volumetric flow rate to e.g. 5 l/h (0.083 l/min) or 20 l/h (0.333 l/min) depending on the planned period of measurement. A sampling time of at least 120 minutes is recommended for determining the mean shift value. The recommended sampling volume is between 5 and 60 l.

The sample should be discarded if the volumetric flow rate at the end of sampling deviates from that set at the beginning by more than ± 5% (DIN 2023).

### 5.2 Sample preparation

The sample carriers must be processed at the latest 7 days after sampling.

The activated charcoal from the adsorption and backup layers of the type B/G tube is placed into separate crimp neck vials and covered with 20 ml of toluene. The vials are sealed and let stand for one hour at room temperature.

The desorption solution (extract made from the adsorption layer) is collected by disposable syringe and a defined application volume (e.g. 6 ml) is placed into a 25-ml volumetric flask. 2.5 ml of the thiophene standard solution (see Sec﻿tion 4.3) are added by bulb pipette and the volumetric flask is then filled to the mark with toluene. 5 ml of this solution are placed into headspace vials and analysed under the conditions specified in Section 6.

To ensure that the range covers the MAK value (16 mg/m^3^), the application of 6 ml of desorption solution is recommended for an air sample volume of 40 l (range about 1.5–15 mg/m^3^). To cover the OELV of 30 mg/m^3^, the application of 3 ml of desorption solution (range about 3.1–31 mg/m^3^) is recommended for an air sample volume of 40 l.

5 ml of the extract prepared from the backup layer are transferred into a headspace vial with a 20-ml disposable syringe and then analysed without ISTD. If carbon disulfide is detected in the backup layer, another sample is analysed after adding the ISTD using the same procedure as for the adsorption layer.

## 6 Operating conditions

The analytical determination is carried out using a gas chromatography system made up of a headspace sampler with a platinum needle, CAP inlet with a deactivated liner and a flame photometric detector under the conditions below.

**Table:** 

**Headspace sampler:**
**Apparatus:**	Turbomatrix 40, PerkinElmer LAS Germany GmbH
**Injection mode:**	time
**Oven temperature:**	90 °C
**Needle temperature:**	100 °C
**Transfer line temperature:**	110 °C
**Transfer line pressure:**	160 kPa
**Thermostating time:**	20 min
**Pressurisation time:**	3 min
**Injection time:**	0.05 min
**Retention time:**	0.2 min
**GC cycle time:**	10 min
**Operating mode:**	constant
	
**Gas chromatograph:**	
**Apparatus:**	Clarus 590, PerkinElmer LAS Germany GmbH
**Column:**	J&W, GS-Q 30 m × 0.53 mm
**Oven temperature:**	150 °C (isothermal)
**Carrier gas:**	helium
**Carrier gas pressure:**	70 kPa (pressure-controlled)
**Injector temperature:**	250 °C
**Detector:**	flame photometric detector
**Detector temperature:**	320 °C
**Range:**	1
**Attenuation:**	0

Carbon disulfide has a retention time of about 1.6 minutes under the described conditions.

## 7 Analytical determination

For the analytical determination, the samples that were prepared as described in Section 5.2 are injected into the gas chromatograph by headspace sampler and analysed in duplicate under the conditions given in Section 6. For this purpose, two headspace vials are prepared and analysed separately. If the resulting concentrations lie above the calibration range, suitable dilutions are prepared and the samples analysed again. A lab blank is analysed according to the same procedure as the analytical samples. For this purpose, 5 ml of the blank solution (see Section 4.3) is transferred into a headspace vial and analysed under the conditions listed in Section 6.

## 8 Calibration

To obtain the calibration function, the calibration samples that were prepared as specified under Section 4.4 are analysed as described in Sections 6 and 7. The resulting quotients of the peak areas of carbon disulfide and ISTD are plotted against the respective concentrations of the calibration samples.

The calibration function is quadratic in the investigated concentration range and should be checked regularly as part of routine analysis. For this purpose, each analytical series must include two control standards with known concentrations for analysis (see Section 4.4.).

A new calibration must be performed if the analytical conditions change or the results of the quality control indicate that this is necessary.

## 9 Calculation of the analytical result

The concentration of the sample solution is determined from the calibration function based on the peak areas. The corresponding mass *X* is determined in mg for each sample from the desorption volume, the application volume and the volume of the sample solution.



(1)

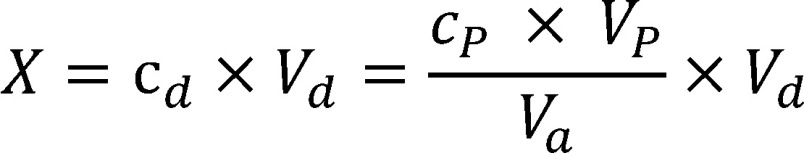




where:

**Table:** 

*X*	is the mass of the substance per sample carrier in mg
*c_d_*	is the concentration of the substance in the desorption solution in mg/l
*V_d_*	is the volume of the desorption solution (in this case 0.02 ml)
*V_P_*	is the volume of the sample solution (in this case 0.025 ml)
*V_a_*	is the application volume in litres
*c_P_*	is the concentration of the substance in the sample solution in mg/l

The mass concentration (*ρ*) is calculated using Equation 2. The analytical result must be adjusted for the recovery *ƞ* of about 70% (see Section 10.2).



(2)

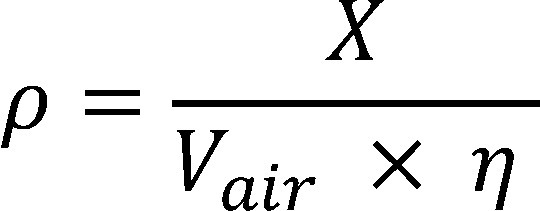




The results are extrapolated to 20 °C and 1013 hPa using Equation 3:



(3)

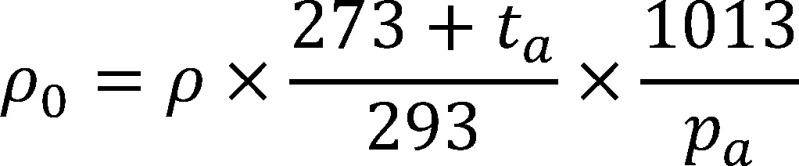




where:

**Table:** 

*ρ*	is the mass concentration of the substance in the air sample in mg/m^3^ at *t_a_* and *p_a_*
*ρ_0_*	is the mass concentration of the substance in mg/m^3^ at 20 °C and 1013 hPa
*V_air_*	is the air sample volume in m^3^ (determined based on the volumetric flow rate and sampling period)
*ƞ*	is the recovery (in this case about 70%)
*t_a_*	is the temperature during sampling in °C
*p_a_*	is the air pressure during sampling in hPa

## 10 Reliability of the method

The characteristics of the method were determined according to the standards DIN EN 482 (DIN 2021 a), DIN EN ISO 22065 (DIN 2021 b) and DIN 32645 (DIN 2008). The method was fully validated. The performance of the method was determined using sample carriers that were first loaded with a test gas with a defined concentration (29.58 mg/m^3^, see Section 4.2). The sample carriers were loaded via a Teflon tube at room temperature (about 25 °C) for 12 min, 120 min or 180 min. A rotameter was placed upstream to maintain a volumetric flow rate of about 0.333 l/min. After 2 hours of sampling, the sample carriers were loaded with the equivalent of 3 mg/m^3^, 30 mg/m^3^ and 45 mg/m^3^, or about one tenth of the currently valid OELV, the OELV and 1.5 times the OELV, respectively. In terms of the currently valid MAK value, the carriers contained the equivalent of 0.2, 2 and 3 times the MAK value, respectively, after 2 hours of sampling. A drum-type gas metre was installed downstream to measure the exact volume.

### 10.1 Repeatability

The repeatability of the method was evaluated using sets of 5 activated charcoal tubes loaded with test gas (nominal concentration 29.58 mg/m^3^). As described in Section 10, the activated charcoal tubes were loaded for 12 min, 120 min or 180 min. The sample carriers were prepared as specified in Section 5.2 and analysed under the operating conditions described in Sections 6 and 7. The coefficients of variation were 6.66%, 4.02% and 2.39%, respectively.

### 10.2 Recovery

The recovery was calculated from the precision data using sets of 5 sample carriers loaded with test gas (nominal concentration 29.58 mg CS_2_/m^3^). The recovery must be determined once for each batch of activated charcoal because it may vary from one batch to another. The recovery for all test concentrations was about 70% and must therefore be accounted for in the results calculation. The recoveries for the three concentrations were 72.1%, 76.3% and 72.8%. After extrapolation to the recovery of 70% that was obtained for the batch of activated charcoal, these values are equivalent to 103%, 109% and 104%, respectively.

### 10.3 Expanded uncertainty

The expanded uncertainty was estimated taking all relevant influencing parameters into consideration as stipulated in ISO 20581 and DIN EN 482 (DIN 2016, 2021 a) and calculated using the Excel tool provided by IFA (IFA n.d.) for the calculation of expanded uncertainty. The main sources of uncertainty with respect to the results obtained by the method as a whole and thus also to the results of the analyses are uncertainties in the sampling procedure (e.g. air sample volume) and in the analytical procedure (e.g. complete desorption, scatter of the calibration function, fluctuations in recovery and reproducibility). The combined analytical uncertainty was calculated to be at most 11%, the expanded uncertainty for the entire method was between 21.2 and 21.8% after applying the expansion factor k = 2.

### 10.4 Influence of humidity

The influence of humidity was determined by loading 5 activated charcoal tubes with test gas (nominal concentration 29.58 mg CS_2_/m^3^) for 12 minutes. Air was then drawn through the tubes for 2 hours at a relative humidity of 80% and a defined volumetric flow rate of 0.333 l/min. The sample carriers were prepared as specified in Section 5.2 and analysed under the conditions described in Sections 6 and 7. A value of 111% was obtained for carbon disulfide after extrapolation to a recovery of 70% for the batch of activated charcoal. At higher levels of humidity, the recovery must be re-evaluated and, if necessary, accounted for using an adjusted correction factor.

### 10.5 Limit of quantification

The limit of quantification was set at the first calibration point because of the quadratic calibration function. The absolute limit of quantification was thus equivalent to 0.5 mg/l. The relative limit of quantification is dependent on the extraction volume and the application volume. The relative limit of quantification for the mean shift value was 0.5 mg/m^3^ at an extraction volume of 20 ml, an application volume of 18 ml and a sample air volume of 40 l. A short-term value of 4 mg/m^3^ was determined based on an air sample volume of 5 l.

### 10.6 Capacity of the adsorbent

The capacity of the sampling medium was evaluated by simultaneously loading 3 activated charcoal tubes with test gas with a concentration of 29.58 mg/m^3^ for 3 hours at room temperature and a volumetric flow rate of 0.333 l/min. This is equivalent to about 1.5 times the currently valid OELV over a sampling period of 2 hours and a sampling volume of 40 l. Less than 5% of the total recovered amount of carbon disulfide was found in the backup layer. As the experimentally determined recovery was about 70%, the recovery in the adsorption layer was 98.5%. Therefore, the capacity of the sampling medium is sufficient also for a sampling period of 3 hours and an air sample volume of 60 l.

If the air sample volume exceeds 60 l or the expected concentrations in air are above 45 mg/m^3^, a second activated charcoal tube should be included to prevent breakthrough.

### 10.7 Storage stability

The storage stability was determined by loading sets of 6 activated charcoal tubes with test gas (29.58 mg/m^3^) for 12 or for 240 minutes at a volumetric flow rate of 0.333 l/min. This is equivalent to concentrations in air of about one tenth and twice the currently valid OELV, respectively, over a sampling period of 2 hours. Three activated charcoal tubes per concentration were analysed immediately. The other three tubes per concentration were analysed after storage for seven days at 1 to 5 °C. The samples were prepared as described in Section 5.2. The values for recovery shown in Table 4 were obtained after analytical determination under the conditions described in Sections 6 and 7 and after taking into consideration the experimentally determined recovery. The loaded sample carriers can be stored for up to 7 days at 1 to 5 °C.

**Tab. 4:** The recovery in terms of storage stability

**Concentration**	**Storage days**	**Recovery**
0.1 OELV	0	95.2%
2 OELV	0	102.3%
0.1 OELV	7	100.1%
2 OELV	7	96.8%

In addition, the possibility of storing the sampling medium in desorption solution was evaluated. For this purpose, the adsorption layer was removed from the loaded tubes and, as described in Section 5.2, immediately covered with toluene. The solutions were stored for 1, 2 and 3 weeks at 1 to 5 °C without undergoing any further processing. Aliquots were then removed and analysed. The recovery decreased to below 80% after only 2 weeks. Therefore, the storage stability for this method is limited to 1 week.

### 10.8 Interference

**Fig. 2:**
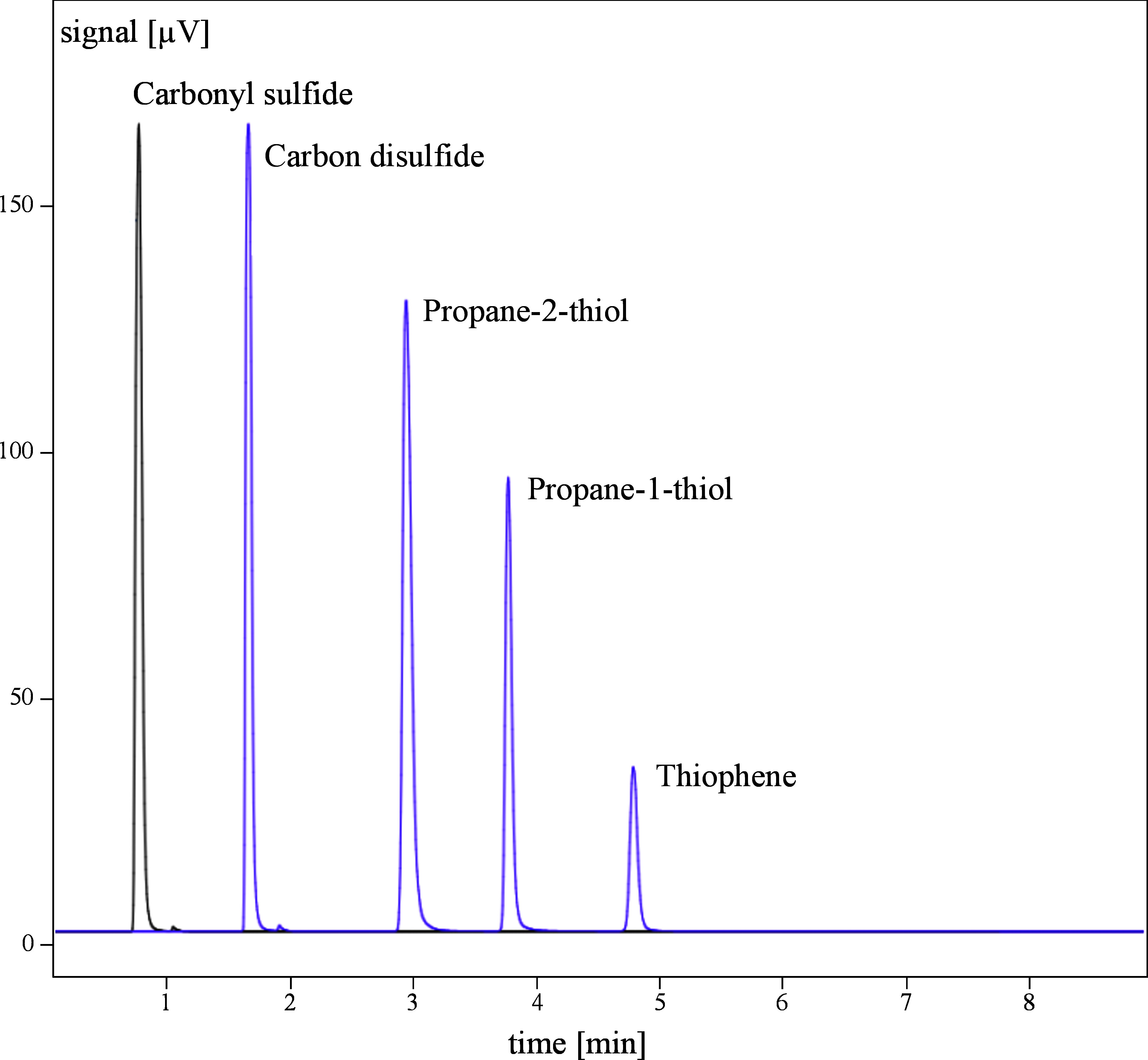
Superimposed gas chromatograms of carbonyl sulfide, propane-2-thiol, propane-1-thiol and thiophene that were used to test for selectivity. Thiophenol could not be detected under the given chromatographic conditions

Potential sources of interference were checked using the substances carbonyl sulfide, propane-1-thiol, propane-2-thiol and thiophenol. No interference was identified under the conditions described in Section 6 (see Figure 2).

### 10.9 Comparative experiments

The method was verified by carrying out comparative experiments with an independent method. Five activated charcoal tubes were loaded with test gas for 120 min as described in Section 10 and prepared and analysed using the method presented here (see Sections 5.2, 6 and 7). Five other activated charcoal tubes were loaded in the same manner, prepared by the company Analytik Service Obernburg GmbH according to an independent protocol and analysed using a photometric method. The values for recovery determined using the two independent methods show good agreement (see Table 5).

**Tab. 5:** Results of the comparative experiments

**Method**	**Nominal concentration** **[mg/m^3^]**	**Determined concentration** **[mg/m^3^]**	**Corrected mean recovery** **[%]**
GC-FPD	30.86	29.80^a)^	96
Photometry	30.86	31.46	102

^a)^ The determined concentration was corrected using a mean recovery of 70% (see Section 10.2).

## 11 Discussion

The analytical method described here is used to determine carbon disulfide in the workplace air in a concentration range from one tenth to twice the currently valid OELV of 30 mg/m^3^. The limit of quantification of the method is low enough to use the MAK value of 16 mg/m^3^ as an assessment criterion. The method was validated at temperatures of 20 to 30 °C. If the conditions at the time of measurement differ significantly from these conditions, the method must be verified accordingly. The method is suitable for monitoring compliance with the STEL. Measurements that were made for comparison purposes using an independent method yielded similar results.
